# Transcriptomic analysis reveals dynamic molecular changes in skin induced by mechanical forces secondary to tissue expansion

**DOI:** 10.1038/s41598-020-71823-z

**Published:** 2020-09-29

**Authors:** Joanna K. Ledwon, Lauren J. Kelsey, Elbert E. Vaca, Arun K. Gosain

**Affiliations:** 1 Division of Plastic Surgery, Northwestern University Feinberg School of Medicine, 225 East Chicago Avenue, Chicago, IL 60611 USA; 2 Department of Plastic and Reconstructive Surgery, Stanley Manne Children’s Research Institute, Ann and Robert H. Lurie Children’s Hospital of Chicago, Chicago, USA

**Keywords:** Gene expression, Next-generation sequencing, RNA sequencing, Cell biology, Medical research, Molecular medicine

## Abstract

Tissue expansion procedures (TE) utilize mechanical forces to induce skin growth and regeneration. While the impact of quick mechanical stimulation on molecular changes in cells has been studied extensively, there is a clear gap in knowledge about sequential biological processes activated during long-term stimulation of skin in vivo. Here, we present the first genome-wide study of transcriptional changes in skin during TE, starting from 1 h to 7 days of expansion. Our results indicate that mechanical forces from a tissue expander induce broad molecular changes in gene expression, and that these changes are time-dependent. We revealed hierarchical changes in skin cell biology, including activation of an immune response, a switch in cell metabolism and processes related to muscle contraction and cytoskeleton organization. In addition to known mechanoresponsive genes (*TNC, MMPs*), we have identified novel candidate genes (*SFRP2*, *SPP1*, *CCR1*, *C2*, *MSR1*, *C4A*, *PLA2G2F*, *HBB*), which might play crucial roles in stretched-induced skin growth. Understanding which biological processes are affected by mechanical forces in TE is important for the development of skin treatments to maximize the efficacy and minimize the risk of complications during expansion procedures.

## Introduction

Skin cells demonstrate an ability to sense mechanical forces and react to a changing environment via a process called mechanotransduction^1^. Applied mechanical forces are translated into cellular signals and transduced inside the tissue, thereby modifying cell behavior and cellular interactions^2^. Mechanical forces can regulate gene expression by direct transmission of the mechanical signal to the nucleolus, or by activation of signaling cascades as a result of conformational changes of proteins^3–7^. These mechanisms allow cells to adapt to changes rapidly and efficiently, preventing harm associated with disturbance in homeostasis. Skin cells, especially fibroblasts, dynamically react to mechanical forces and undergo transformations to adapt to the changing environment^8–11^. Numerous studies have demonstrated that mechanotransduction plays a significant role in many aspects of tissue growth and repair, such as cell proliferation^7,12–14^, cell differentiation^7^, extracellular matrix (ECM) remodeling and homeostasis maintenance^15–18^. It has been shown that when mechanical forces are applied to skin, the established state of cytoskeletal elements shifts and the position of all connected molecules and organelles are affected, changing the course of reactions that occur and resulting biochemical signals^19^. Altogether, these features give skin an extraordinary ability to grow in response to mechanical stimulation^20^, making tissue expansion (TE) ideal for obtaining extra skin in plastic and reconstructive surgery^21–25^. Importantly, the biomechanical properties of stretched skin remain unchanged^26^ and, as we previously reported, the initial increase in epidermal thickness normalizes over time^27^. Although TE is broadly used in clinical settings^28–30^, complications are still very common^31–33^ and molecular mechanisms involved in mechanically-induced skin growth are not well understood. Subsequently, this medical procedure does not include any approved skin pretreatment and the procedural technique is highly dependent on the experience and subjective assessment of the surgeon. Thus, to reduce the risk and improve the efficacy of the procedure, the evaluation of global changes in gene expression during TE is necessary to discover key molecular pathways involved in skin growth and regeneration stimulated by mechanical forces.

In this study, we have revealed strong activation of an immune response after 1 h of TE, and detected disruption in processes related to metabolism, cell contraction and cytoskeleton rearrangement between 24 h and 7 days of TE. Moreover, we have identified hub genes for each protein–protein interaction (PPI) network and reported new potentially mechanoresponsive genes. Overall, our results provide key insight regarding biological processes affected by mechanical forces in TE, all of which work to simultaneously induce skin growth and maintain tissue homeostasis and integrity.

## Results

### Study design and overview

**Figure 1:**
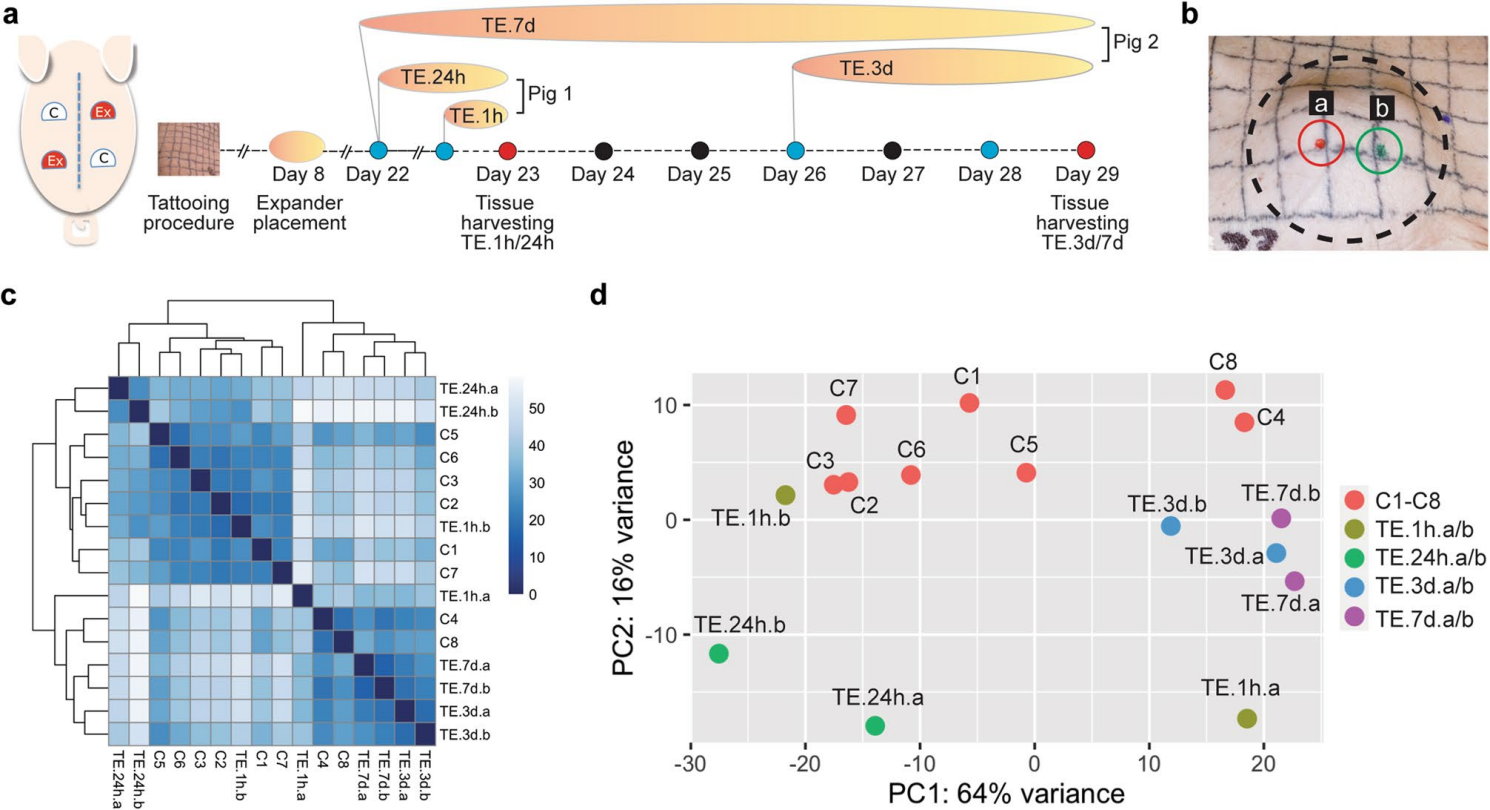
Overview of the study design and presentation of overall similiarity among control and expanded skin samples. (**a**) Schematic presentation of expander placement (Ex) and control (C) locations on the porcine model and procedural timeline of skin expansion for 1 h (TE.1h), 24 h (TE.24h), 3 days (TE.3d) and 7 days (TE.7d). (**b**) Representative image of samples location on the apex (“a) and middle (“b) of the expander, demonstraiting close proximity of the “a” and “b” areas from which biopsies were taken. The dashed circle indicates the periphery of the expander. (**c**) HeatMap of Eucliden sample distances with unsupervised hierarchical clustering and (**d**) Principal Component Analysis (PCA) using the rlog-transformed values for control (C1–C8) and expanded samples collected from the apex/middle of the expander (TE.1h.a/b, TE.24h.a/b, TE.3d.a/b, TE.7d.a/b).

To better understand the molecular response of skin following TE, we performed an in vivo study using a porcine TE model^34^ and evaluated the skin transcriptome profile at 1 h (TE.1h), 24 h (TE.24h), 3 days (TE.3d) and 7 days (TE.7d) of expansion (Fig. 1a). The long timeframe allowed us to assess transcriptional changes in acute—at 1 and 24 h—and prolonged—at 3 and 7 days—periods of stretch. In order to detect global changes in gene expression, we performed RNA-seq analysis for 16 pooled-samples, eight controls and two tested samples per timepoint. Tested samples were located in close proximity to each other, on the apex (“a”) and middle (“b”) of the expander. Samples “a” and “b” were treated as replicates because they were collected from the expanded skin at the same timepoint (Fig. 1b). On average, we generated 2,150,192 reads per sample, ranging from 1,444,230 to 2,578,869 reads. Unsupervised hierarchical clustering of the sample distances presented by heatmap showed high similarities between control samples (six out of eight controls grouped together), and clear separation of TE.24h samples from other TE samples. Surprisingly, sample TE.1h.b, collected from the middle of the expander, had a shorter sample distance to the control than to the other TE samples (Fig. 1c), indicating that after 1 h of TE, skin at the apex was more strongly affected than skin in the middle of the expander. This suggests that the molecular response of skin in TE is most pronounced at the apex of the expander where the strengths of mechanical forces are the highest, and over time extends to the whole tissue. The PCA analysis supports these observations (Fig. 1d).

### Differential gene expression analysis

**Figure 2:**
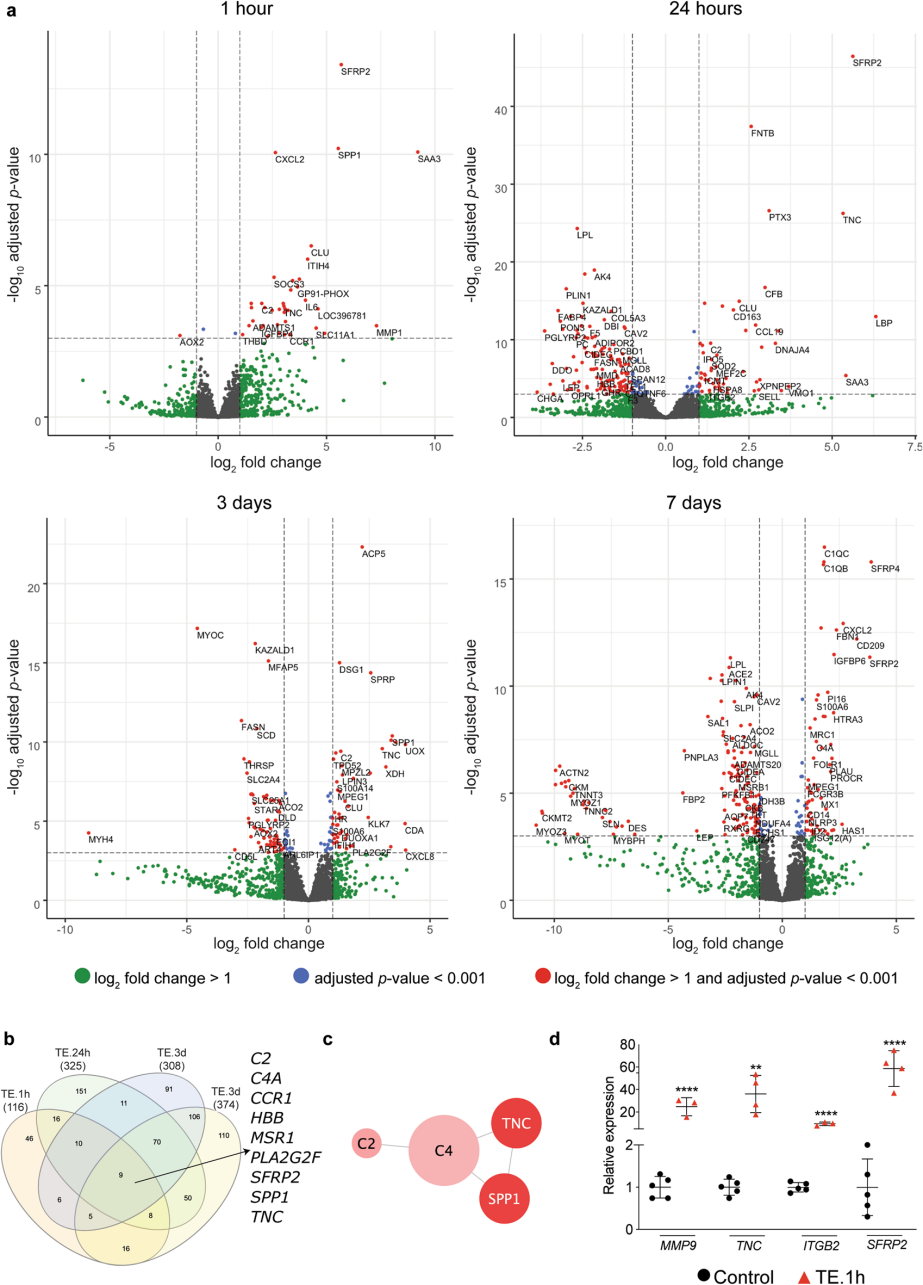
RNA-seq analysis revealed numerous changes in gene expression throughout the duration of expansion. (**a**) Volcano plots showing all mapped genes at different timepoints of TE, with the left and right sides presenting downregulated and upregulated genes, respectively. (**b**) Venn diagram of the comparative analysis of DEGs from 1 h (TE.1h), 24 h (TE.24h), 3 days (TE.3d) and 7 days (TE.7d) of expansion, revealing nine genes common to all conditions. (**c**) Graphical presentation of STRING analysis showing interactions between overlapping genes reported in (**b**). (**d**) Relative expression of selected genes assessed by qRT-PCR in skin biopsies after 1 h of expansion compared to unexpanded controls (n ≥ 3) confirming the accuracy of results obtained by RNA-seq analysis. The values of qRT-PCR were normalized to *B2M* expression. Error bars represent SD. Statistical significance are shown as **P ≤ 0.01, ****P ≤ 0.0001.

A distinct pattern of gene expression was observed in the stretched skin samples compared to the unexpanded control at each tested timepoint during the 7-day period of skin expansion. By analyzing the RNA-seq data with the criterion of |log_2_FC|> 1 and adjusted *p*-value < 0.05 we have identified significant changes in the expression of 116 genes (104 upregulated, 12 downregulated) at 1 h, 325 genes (118 upregulated, 207 downregulated) at 24 h, 308 genes (108 upregulated, 200 downregulated) at 3 days, and 374 genes (111 upregulated, 263 downregulated) at 7 days, compared to their respective unexpanded controls (Fig. 2a, Fig. S1a). Starting at 24 h, the number of downregulated genes was significantly higher than upregulated genes. Moreover, the greatest number of differentially expressed genes (DEGs) was reported at 7 days of TE, indicating increasing changes in molecular responses throughout the expansion period.

The comparative analysis of DEGs revealed a low degree of overlap between all tested timepoints, ranging from 2 to 8% (Fig. 2b). A significant portion of DEGs was unique and accounted for 40% (46 genes), 46% (151 genes), 30% (91 genes) and 30% (110 genes) of DEGs identified at 1 h, 24 h, 3 days and 7 days of TE, respectively, indicating that TE induces wide-range time-dependent molecular changes. The highest similarity between transcriptome profiles was observed for 3 and 7 days of TE, showing 190 overlapping genes. These results indicate that mechanical forces altered the transcription profile of the skin as early as 1 h after the initiation of the procedure, and homeostasis had not been reached even after 7 days. Regardless of timepoint, the expression of nine genes: *SFRP2*, *SPP1*, *TNC*, *CCR1*, *C2*, *MSR1*, *C4A*, *PLA2G2F*, *HBB* was significantly changed in the stretched skin (Fig. 2b), suggesting that these genes might be crucial in skin remodeling triggered by an altered mechanical environment. Seven of them (*SFRP2*, *SPP1*, *TNC*, *CCR1*, *C2*, *MSR1*, *C4A*) were consistently upregulated. *PLA2G2F* was downregulated at 1 and 24 h, and upregulated at 3 and 7 days. *HBB* was upregulated at all timepoints except 7 days. The Retrieval of Interacting Genes database (STRING) showed well-established interactions between *TNC*, *SPP1*, *C4*, *C2* (Fig. 2c), although the character of the interactions is unknown. Additionally, we have selected *SAA3, ARG1, SLC11A1, CLU, CECR1, C7, CFB* (consistently upregulated) and *AOX2*, *KAZALD1* (consistently downregulated) between 1 h and 3 days of TE as potentially important mechanosensitive genes. These candidates are worth future investigation in the context of mechanoresponsive genes, given their rapid and strong activation after mechanical stimulation*.* The qRT-PCR analysis for selected genes confirmed the accuracy of RNA-seq results (Fig. 2d, Fig. S1b).

### Gene ontology (GO) enrichment pathway analysis

**Figure 3:**
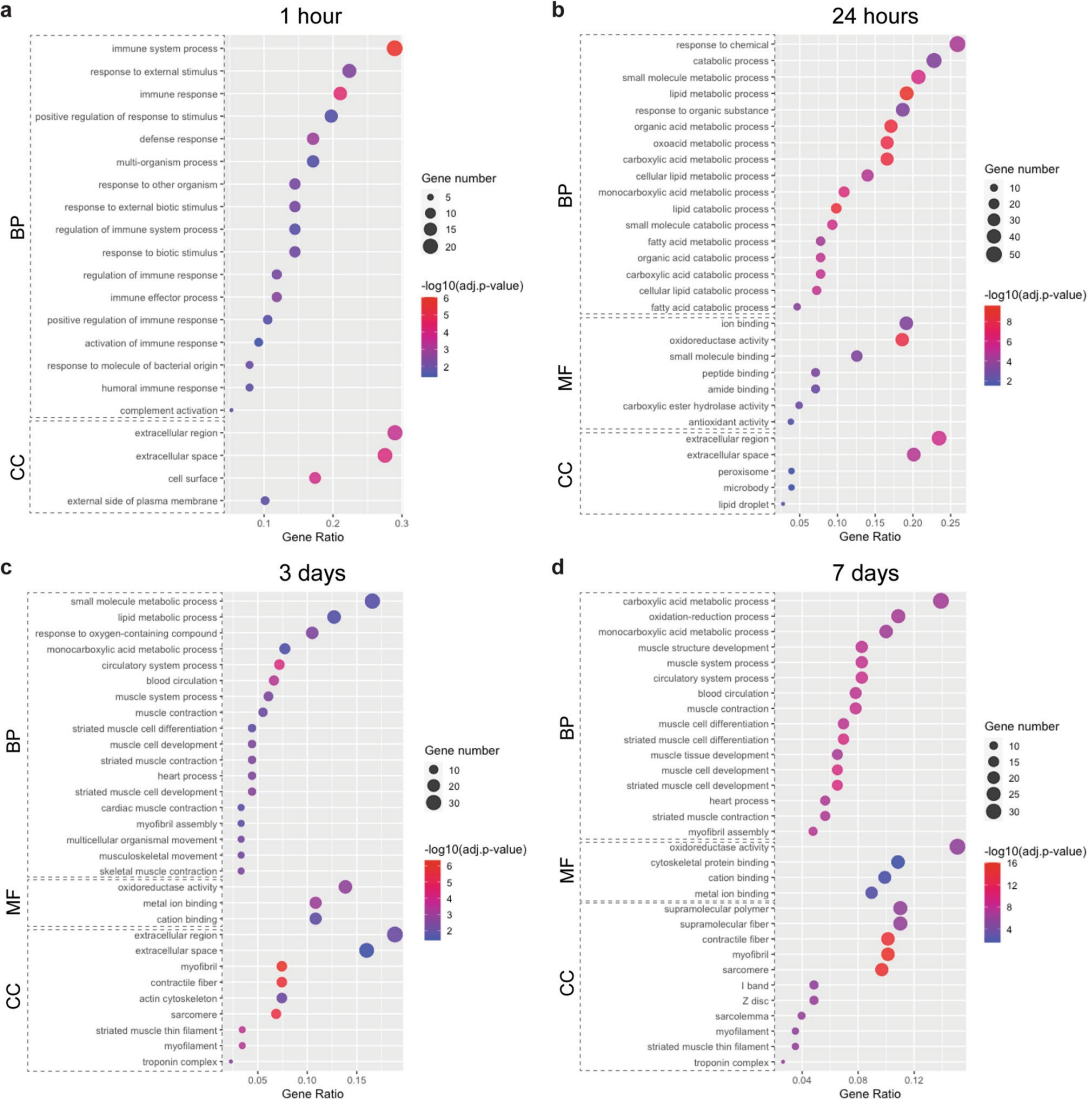
GO enrichment analysis showed evolving changes in the molecular response of skin during TE. Dot plots of representative GO terms at (**a**) 1 h, (**b**) 24 h, (**c**) 3 days and (**d**) 7 days, showing the diversity of affected processes. Gene ratio indicates the number of DEGs associated with the GO term divided by the total number of DEGs. GO terms were ordered according to the gene ratio. The size of the dots represents the number of DEGs associated with the GO term and the color represents the negative value of log_10_ of the adjusted *p*-value (adj. *p*-value). *BP* biological processes, *MF* molecular functions, *CC* cellular components.

**Figure 4:**
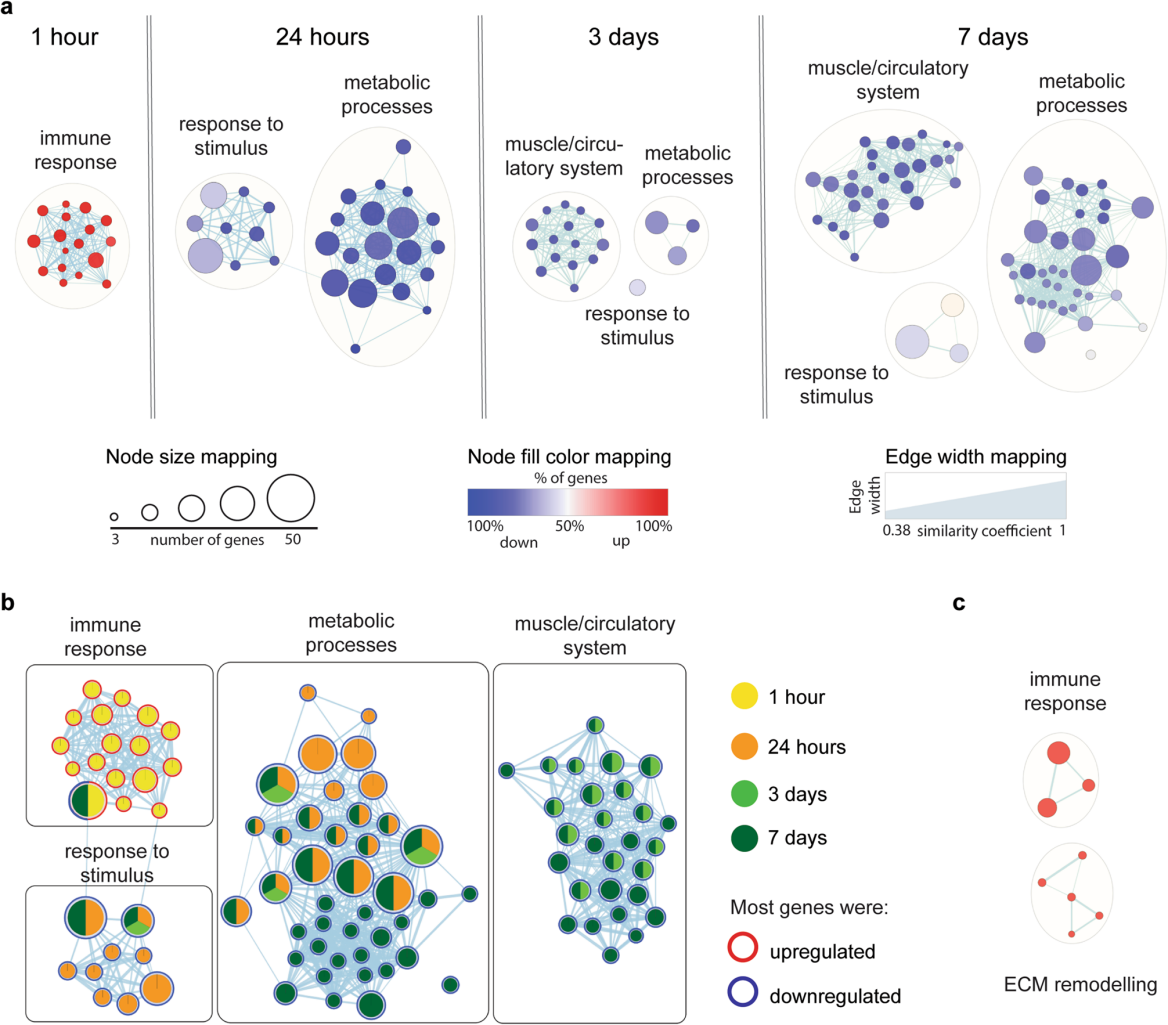
Enrichment maps depicted the progressive shift in dominant biological processes throughout the duration of expansion. (**a**) Enrichment map of significantly enriched biological processes revealed by GO analysis at each tested timepoint of expansion. (**b**) Enrichment map of REAC pathway analysis at 1 h of expansion. (**c**) Cohesive view of enriched biological processes highlighting at which timepoints of expansion their components are particularly active or dampened. For all panels, the enrichment map arranges biological processes as a similarity network. In the network, each node represent a gene-set associated with that biological process. Edge thickness is proportional to the overlap between the gene-sets. Nodes are colored according to the percentage of upregulated and downregulated genes associated with this node.

To elucidate biological processes and molecular pathways with significant correlation to skin growth and regeneration from TE, GO analysis—including three functional categories such as biological process, molecular function and cellular component—was performed. The results were expanded for analysis using the Reactome (REAC) database and were reported only if additional information was discovered. The enrichment analysis of DEGs at 1 h of TE showed that most of them were associated with inflammatory processes, such as response to external stimulus, immune response or defense response (Fig. 3a, Table S1). All of the genes but one (*PLA2G2F*) were upregulated and *SAA3, CXCL8, SLC11A1, CLU* showed the highest increase in expression (|log_2_(FC)|> 4) (Table S2). This indicates that inflammatory processes were immediately triggered in response to mechanical forces from the tissue expander. The EnrichmentMap and AutoAnnotate plugins in Cytoscape showed strong interconnection between all GO terms (Fig. 4a,b). Further analysis using the REAC database also revealed a cluster with genes linked to ECM remodeling (*MMPs*, *TIMPs*, *IGFs*), confirming the crucial role of this pathway in early stages of skin molecular response to mechanical stress (Fig. 4c).

At 24 h of TE, a vast majority of DEGs were associated with metabolic processes, including catabolism of lipids and organic acids, followed by genes involved in response to stimuli (hormones, chemicals or oxygen-containing compounds) (Fig. 3b, Table S3). All enriched processes were visualized in Cytoscape and divided into two clusters using Markov Cluster (MCL) algorithm (Fig. 4a,b). The most upregulated genes were *LBP, SAA3, ARG1*, while the most downregulated were *CYP2A19*, *STAR, CHGA* (|log_2_(FC)|> 3.9) (Tables S4, S5). These results indicate that there are significant changes in the energy pathways and processes related to the response to stimulus in the stretched skin at an early stage of TE.

The enrichment analysis of DEGs at 3 days of TE revealed their association with processes related to the circulatory system, muscle, metabolism and response to oxygen-containing compounds (Fig. 3c, Table S6). The most significantly enriched GO term was “circulatory system process”, followed by “blood circulation”, whereas the most common GO terms were related to muscle development and contraction. Based on the MCL algorithm, processes related to the circulatory system and muscle were clustered together (Fig. 4a,b). Among genes related to the muscle/circulatory system, all genes but one (*ID2*) were downregulated and the highest decrease in expression was observed for *TNNT1, MYH7* and *CASQ1* (|log_2_(FC)|> 5). In the cluster with metabolic processes, the vast majority of genes (32 of 41 genes) were downregulated, of which *AMPD1, ACLY* and *PLCD4* showed the highest decrease in expression, whereas *ARG1, CDA* and *XDH* were the most upregulated (|log_2_(FC)|> 3). Lastly, among genes related to response to oxygen-containing compounds, *CXCL8, LBP* and *SLC11A1* were the most upregulated, while *TPM1* was the most downregulated (|log_2_(FC)|> 3) (Tables S4, S5, S7).

At 7 days of TE, the alterations in processes associated with the muscle/circulatory system and metabolism still persisted, but the number of classified processes in these two clusters had significantly increased by this timepoint, showing 28 and 35 GO terms, respectively (Figs. 3d, 4a,b, Table S8). The muscle/circulatory system cluster included processes involved in muscle development and contraction, and processes related to blood circulation, similar to 3 days of TE. In the cluster linked to metabolism, alongside processes that were also enriched at TE.24 h and TE.3d, we reported processes related to energy production (glycolysis, gluconeogenesis, ATP generation) and biosynthesis. Moreover, our results showed enrichment for processes such as response to oxygen-containing compounds and response to chemicals. Notably, regardless of biological process, a significant proportion of DEGs was downregulated. The most downregulated muscle-related genes were *ACTN2, TNNT3* and *TNNC2*, metabolic-related genes were *CKMT2, CKM* and *FITM1*, and *CKMT2, ACTN2* and *MYOT* among genes related to response to stimulus (|log_2_(FC)|> 7). Among the muscle-related genes, besides *ID2* whose increased expression was also reported at TE.3d, an additional two genes *FOS* and *KCNE3* were upregulated. Furthermore, genes *HAS1, SOCS3* and *XDH* were the most upregulated among metabolism-related genes (|log_2_(FC)|> 1.7), while *GAP43, CXCL2* and *HAS1* were the most upregulated among genes related to response to stimulus (|log_2_(FC)|> 2.6) (Tables S4, S5, S7). Altogether, these results indicate that an immune response and response to stimuli play a significant role in the molecular response of skin at the beginning of tissue expansion, whereas processes related to muscle, blood circulation and metabolism are predominantly involved in skin growth and regeneration at later stages of TE.

In the category of molecular function, the enrichment analysis did not show any significant results for DEGs at 1 h of TE. However, the analysis at TE.24h, TE.3d, and TE.7d revealed that DEGs, regardless of time of expansion, were involved in catalytic activity and molecule binding functions. The catalytic activity involved enzymes such as oxidoreductase and carboxylic ester hydrolase, whereas the molecule binding function included ion, peptide or cation binding. In addition, the antioxidant activity function was detected exclusively at 24 h, whereas the cytoskeletal protein binding function was only at 7 days of TE (Fig. 3a–d).

Extracellular space and extracellular region were the most commonly represented cellular components for DEGs at 1 and 24 h of TE. In addition, cellular components such as cell surface and external side of plasma membrane were enriched at 1 h, while lipid droplet, peroxisome and microbody were enriched at 24 h of TE. The most common cellular components represented for DEGs at 3 and 7 days of TE included muscle compartments such as myofibril, contractile fiber or actin cytoskeleton. Extracellular space was also significantly enriched at 3 days, while cytoplasm and supramolecular complex were exclusively enriched at 7 days of TE (Fig. 3a–d).

### Kyoto encyclopedia of genes and genomes (KEGG) enrichment pathway analysis

**Figure 5:**
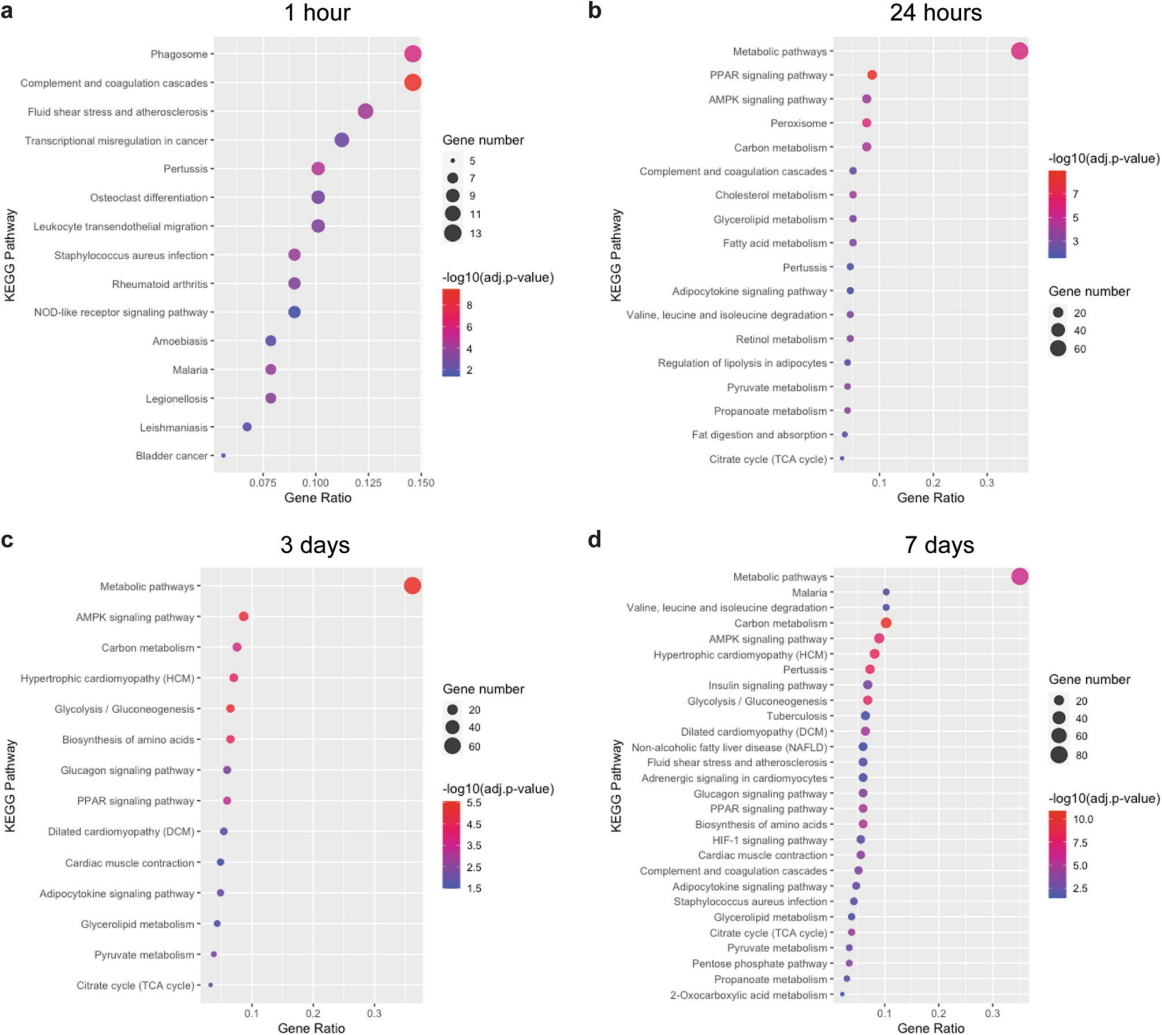
KEGG pathway analysis demonstrated a shift in processes that were enriched, from immune response pathways to metabolic and muscle-related pathways during expansion. Dot plots of enriched pathways at (**a**) 1 h, (**b**) 24 h, (**c**) 3 days and (**d**) 7 days of expansion. Gene ratio indicates the number of DEGs associated with the KEGG term divided by the total number of DEGs. KEGG terms were ordered according to the gene ratio. The size of the dots represents the number of DEGs associated with the GO term and the color represents the negative value of log_10_ of adjusted *p*-value (adj.*p*-value).

To further investigate the molecular pathways affected by mechanical forces from TE, we performed KEGG pathway analysis. A total of 15, 18, 14 and 28 significantly changed pathways were identified at TE.1h, TE.24h, TE.3d, and TE.7d, respectively (Fig. 5a–d). KEGG analysis for DEGs at 1 h showed enriched pathways predominantly related to immune response (complement and coagulation cascades, phagosome, NOD-like receptor signaling pathway) and human diseases (cardiovascular, infectious, immune and cancer diseases.) Significantly more DEGs also overlapped with the osteoclast differentiation pathway, which is part of the development and regeneration class (Fig. 5a). At 24 h of TE, the most enriched and common pathways were related to metabolism and energy production, including peroxisome proliferator-activated receptor (PPAR) and AMP-activated protein kinase (AMPK) signaling pathways. In contrast, immune response pathways were in the minority (Fig. 5b). KEGG analysis of DEGs at later stages of TE, 3 and 7 days after expander inflation, revealed pathways mainly related to metabolism and cardiac muscle, including cardiovascular diseases (Fig. 5c). Among the most enriched metabolic pathways were AMPK, PPAR, and Glycolysis/Gluconeogenesis. While at 3 days cardiac muscle contraction was the only pathway related to muscle, at 7 days the HIF-1 signaling pathway was also enriched. Moreover, at 7 days the complement and coagulation cascades, as well as pathways related to infectious and metabolic diseases were mapped (Fig. 5d). These results suggest that those pathways may play crucial roles in the skin growth induced by mechanical forces.

### The time-related PPI networks in TE

**Figure 6:**
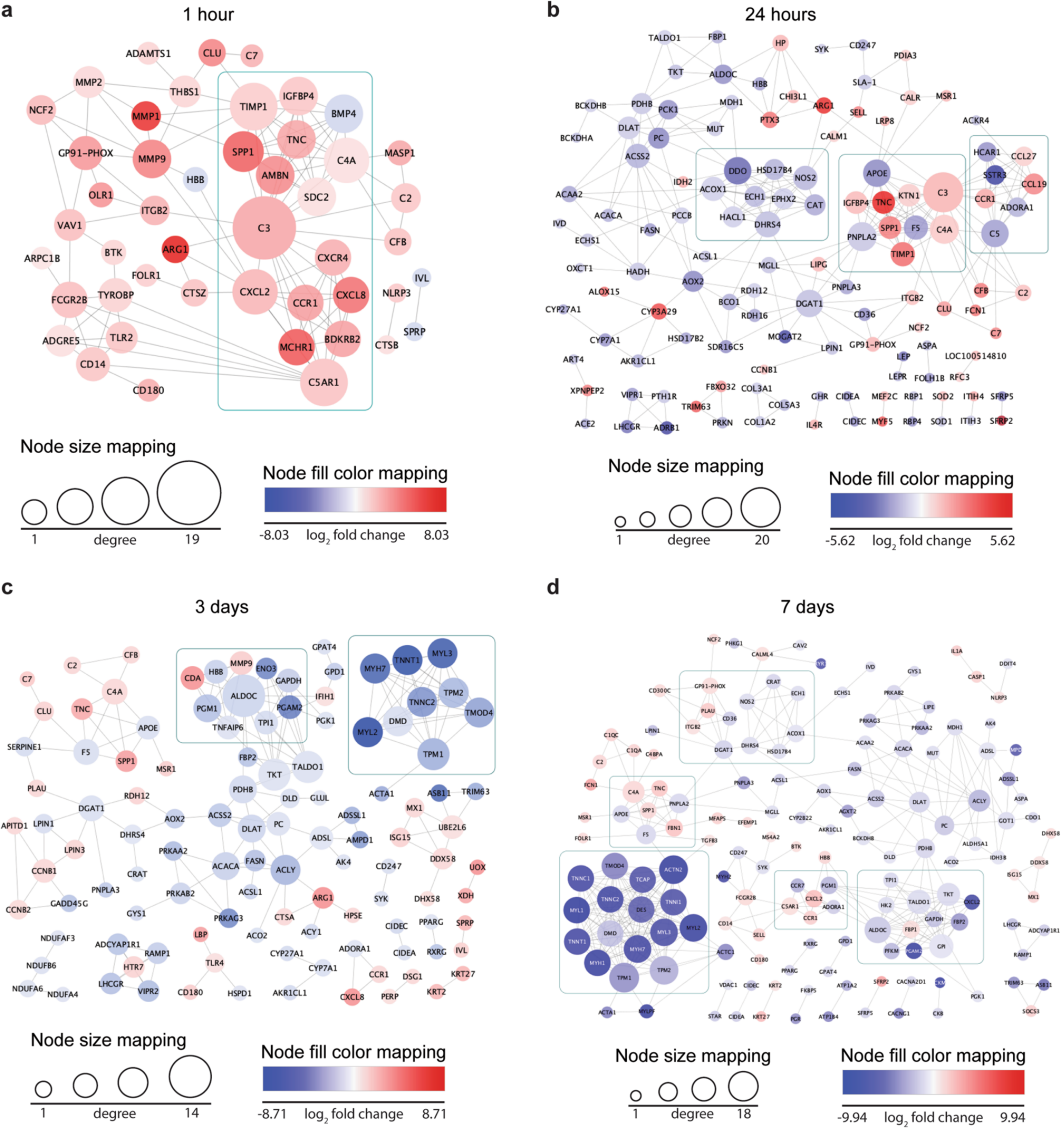
PPI network analysis revealed the complexity of DEG interactions and molecular responses involved in skin growth and regeneration stimulated by mechanical forces from expansion. Graphical presentation of DEGs (nodes) at (**a**) 1 h, (**b**) 24 h, (**c**) 3 days, (**d**) 7 days, where the boxed areas represent highly interconnected genes identified by MCODE algorithm.

The PPI network at 1 h of TE, constructed using STRING web tool and visualized in Cytoscape, consisted of 47 nodes and 126 edges. Using the MCODE application in Cytoscape, we revealed one significant gene module which included 16 genes, of which all but one (*BMP4*) were upregulated (Fig. 6a). By using NetworkAnalyzer tool in Cytoscape, genes *C3*, *C5AR1*, *TIMP1*, *CXCL2*, *C4A*, *MMP9* were classified as the hub genes. This data highlights the prominent role of the complement system, chemokines and MMPs in the inflammatory phase activated by mechanical forces from TE. The PPI network constructed for DEGs at 24 h of TE was comprised of 124 nodes, 272 edges and three gene modules (Fig. 6b). The following genes met the requirements to be considered hub genes: *C3, PNPLA2, DDO, APOE, DGAT, DHRS4, ACSS2, NOS2, ACOX1, AOX2, PDHB*. At 3 days of TE, among 114 nodes and 195 edges, two modules were identified and *ALDOC, ACLY, ACACA, DLAT, PDHB, DGAT, PC, ADSL, ACSS2* were classified as the hub genes (Fig. 6c). Interestingly, the PPI network constructed for DEGs at 7 days of TE was the most complex, comprised of 153 nodes, 358 edges and five modules. The following genes were classified as the hub genes: *ACTN2, ALDOC, GPI, TALDO1, TKT, ACLY, C4A, DLAT, F5, PNPLA2, PC, PDHB, DGAT1, ADSL, CXCL2, DHRS4, APOE, ACSS2, ACACA, FBN1, ACOX1, GOT1, PGM1* (Fig. 6d). These results clearly demonstrate that at each analyzed timepoint, a significant proportion of proteins encoded by DEGs interact with each other. The presence of different modules at each timepoint confirms that DEGs in TE are involved in multiple biological processes, in which the identified hub genes might play a crucial role.

## Discussion

TE is a common reconstructive modality for various clinical conditions, including craniofacial defects^25^, burn scars^35^, and giant congenital nevi^36^. TE is also used for reconstruction following ablative cancer surgery, and it is the most common form of breast reconstruction following mastectomy for breast cancer^37^. However, the biological aspects of TE are still under investigation, which limits the ability to include science-based information in implementing skin pre-treatment for such procedures. Previously, we found that a potential disturbance in epidermal structure caused by excessive stretch-induced keratinocyte proliferation is limited by elongation of the basal layer and an increase in the number of rete ridges^38^. Here, we describe the gradual activation of widespread transcriptional changes in skin induced by mechanical forces during TE. Based on the gene expression patterns, three different phases of the skin’s molecular response can be distinguished, including activation of an immune response, regulation of metabolic processes, and downregulation of cell contractions. The activation of an immune response was especially distinct at 1 h of TE, while disruption of metabolic and muscle related pathways started at 24 h and lasted until 7 days of TE with varied intensity.

The pairing of a porcine TE model with RNA-seq analysis for multiple timepoints allows us to provide a comprehensive list of DEGs in skin following TE. Given the high similarity between porcine and human skin, including histological, physiological and immunological properities^39,40^, we are assured that the obtained results may reflect, to a great extent, the molecular changes in human skin from expansion procedures performed in clinical settings. The presented results confirmed that during TE, skin dynamically responds to mechanical forces by changing its gene expression profile. We revealed a distinct gene expression pattern for each tested timepoint, demonstrated by a small proportion of overlapping genes. In addition to known mechanoresponsive genes such as *TNC, MMPs, TIMP1* we have identified others such as *SAA3, ARG1, SFRP2, SPP1, SLC11A1, CLU, CCR1, CECR1, C7, MSR1, CFB, C2, C4A, KAZALD1, HBB, PLA2G2F, AOX2*, that have not yet been correlated with response to mechanical forces. However, our report of differential expression of these genes for at least three days after initiation of TE indicates that it may be valuable to investigate their role in the mechanotransduction process. Of notable interest are genes *SFRP2*, which enhances the osteogenic differentiation of apical papilla stem cells by antagonizing the canonical WNT pathway^41^, and *PLA2G2F*, which regulates skin homeostasis and keratinocyte differentiation^42,43^. Additional genes include *SPP1,* which is involved in post-injury tissue remodeling in mice^44^, and *SAA3*, which regulates collagenase expression an in vitro in rabbit fibroblast, substantiating their roles in tissue remodeling^45^.

Based on GO and KEGG analysis we revealed a strong activation of inflammatory processes and an increase in activity of genes involved in ECM remodeling in the skin as early as 1 h after expansion was initiated. Simultaneously, as we previously reported, from 1 h to 3 days of TE, we observed a gradual increase in keratinocyte proliferation in the stretched skin^27^. Our results are in agreement with studies showing the importance of an immune response in stretch-induced skin regeneration in rats^46^ and in stimulating excessive keratinocyte proliferation in psoriasis^47^. Therefore, our results confirm that a controlled, moderate inflammatory response activated by mechanical forces is an important factor in stimulating keratinocyte proliferation and initiation of skin regeneration. Accordingly, our analysis revealed that components of an immune response such as *CXCL8, SAA3, CLU* and *C3*, which stimulate cell proliferation in vitro and in vivo^48–54^, might be also involved in keratinocyte proliferation in the mechanically stimulated skin. The significance of these findings in relation to skin growth is supported by a study showing that *CXCL8* also regulates keratinocyte migration^55^. Interestingly, the persistent upregulation of *CCR1* during TE suggests that this chemokine receptor might play a significant role in skin regeneration induced by stress, although apart from its role in re-epithelization^56^ and strong activation after skin injury^57^, little is known about its biological function. In addition, increased expression of *ARG1*, a component of metabolic pathways that was highly activated between 1 h and 3 days of TE, has been linked to keratinocyte hyperproliferation in psoriasis^58^. The authors proposed that overexpression of *ARG1* limits production of nitric oxide, an inhibitor of cellular proliferation^58^. Furthermore, *ARG1* is activated in tissue remodeling processes, as demonstrated by studies on fibrotic lung disease^59^ or allergic asthma^60^. *ARG1* also promotes wound healing and its inhibition was correlated with increased inflammation^61^. Therefore, we hypothesize that *ARG1* activation in the stretched skin might play a significant role in remodeling processes.

Our analysis at 1 h of TE also revealed early activity of factors related to tissue remodeling (*MMPs, TIMPs, IGFBP4*) and the PPI network shed light on the interactions between chemokines, cytokines and proteolytic processing factors in the inflammatory response induced by mechanical forces. Among the hub genes were genes involved in immune response (*C3, C4A, C5AR1, CXCL2*) and in tissue remodeling processes (*TIMP1*, *MMP9*), confirming this observation. Similar interplay has been shown between MMP-2, MMP-9 and leukocytes at the blood brain barrier, where MMPs stimulate leukocyte migration controlled by chemokines^62^. Moreover, in skin wound healing and regeneration, it has been shown that macrophages play a large role in restoring tissue homeostasis, in part by remodeling the ECM and synthesizing cytokines and growth factors^63^. Our results are in agreement with studies showing the importance of *MMP9* and *TIMP1* in inflammation and breakdown of the ECM during tissue remodeling^64^. The GO analysis focused on cellular components confirmed that in early stages of TE, many processes take place in the ECM where mechanical pressures immediately force physical changes in the cellular space, and a biomolecular cascade occurs to reinstate skin homeostasis. Our results underline the importance of inflammatory processes in the initial skin response to stretch, however**,** a better understanding of how immune system activation aids skin growth induced by mechanical stretch requires additional functional studies.

In contrast, at 24 h of TE, GO and KEGG analysis showed notably less emphasis on immune pathways, instead shifting to metabolism and stimulus-driven processes. Interestingly, the disturbances in pathways involved in response to stimulus, which still included components of immune response, persisted until 7 days of TE. The altered complement and coagulation cascades and broad changes in expression of cytokines and their receptors during the entire TE procedure indicate that an immune response also plays a significant role in later stages of skin remodeling. Although the most differentially expressed metabolism-related genes at 24 h of TE are not known to be crucial in tissue remodeling, it has been shown that metabolic processes, like glycolysis and fatty acid oxidation metabolism, play a role in regulating inflammation and ECM deposition^65^. Thus, the changes in metabolic processes in early stages of TE may have occurred to modulate the immune response triggered immediately by mechanical forces. The enriched metabolic processes included lipid metabolism, PPAR and AMPK signaling pathways and the TCA cycle. Additionally, the PPI network constructed for DEGs at 24 h of TE provided insight into the interdependence between identified DEGs. A high number of hub genes indicates its proportional influence in the network. Most of the genes involved in PPAR signaling, including transcription factor *PPARG* and downstream target *ACOX1*, were downregulated in the stretched skin at 24 h and 7 days, suggesting a sustained decrease in activity of the PPAR pathway during TE. Similar observations were made for genes related to the TCA cycle. Our results are in agreement with another in vitro study which showed downregulation of genes involved in catabolic processes in human mesenchymal stem cells subjected to stretch^66^. Similarly, in vitro studies on dermal primary human fibroblasts showed decreased expression of genes involved in the PPAR pathway in hypoxic conditions, resulting in increased ECM synthesis^67,68^. Moreover, another recent report revealed a direct negative correlation between PPAR and TCA pathway activity and deposition of ECM in a murine model of fibrosis^65^. It is well documented that tensile stress induces intensified ECM synthesis and remodeling^69^. Therefore, we assume that the revealed changes in the activity of metabolic pathways result from an intensive production of ECM. Notably, *ACOX1* was identified as a hub gene in the PPI networks constructed for DEGs at 24 h and 7 days, confirming its significance in processes related to tissue expansion during TE. In contrast, the activation of AMPK pathway in response to mechanical force has previously been reported^70^. However, although our analysis at TE.24 h showed increased expression of genes coding regulatory units of AMPK (*PRKAA2*, *PRKAA2*, *PRKAG3*, *PRKAB2*, *PFKM*), these results were not statistically significant, and by 7 days of TE all of these genes were significantly downregulated. Based on GO and KEGG analysis, the metabolic processes exclusively enriched at 7 days of TE involved energy-consuming biosynthetic pathways, including synthesis of amino acids and carbohydrates. Among the most activated genes at this timepoint was *SOCS3*, whose role in epidermal homeostasis has been highlighted in recent studies conducted on mice and human HaCaT cells^71^. Altogether, our results clearly present that alterations in metabolic pathways seen at 24 h of TE gradually evolve, and a number of DEGs involved in metabolism increase over time, creating an expansive list of metabolic processes affected during TE. This demonstrates the importance of such metabolic transitions in skin cells’ response to mechanical forces in TE in order to adapt and re-establish tissue homeostasis.

The importance of mechanical forces in skin physiology and the pathogenesis of skin diseases is unquestionable^15,72,73^. Fibroblasts, through a mechanotransduction process, dynamically react to external stimuli, including tensile stress, not only by increasing production of ECM^69^ but also by regulating cell-mediated contractions^10^. GO analysis focused on biological processes and cellular components clearly showed that numerous identified DEGs at 3 and 7 days of TE were involved in muscle-related processes, including muscle contraction. Starting at 3 days of TE, we observed a significant decrease in expression of muscle-related genes, mainly involved in muscle contraction, including troponins (*TNNT1, TNNT3, TNNC2, TNNI2*), myosins (*MYH7*), and members of the tropomyosin family (*TPM1, TPM2*), which strongly indicates a decrease in actin-myosin regulated cell contractions. Analysis of studies on different animal species provides convincing proof that scarless spontaneous wound regeneration is possible, mainly because of an absence of wound contraction^74^. Since TE stimulates skin regeneration and restored tissues maintain their biomechanical features, similarities in the molecular activity between these two processes are likely. Our results indicate that the inhibition of cell contraction during TE might be one of the mechanisms used by cells to successfully adapt to stretch and maintain tissue integrity. The critical role of cytoskeleton rearrangement as a first line of cells’ defense against disturbance in homeostasis caused by external mechanical forces has been elucidated^2,75^. In TE procedures, the magnitude of the mechanical forces experienced by the tissue will vary, as the skin phases through stress (after expander inflation) and rest periods. It has been shown that an increase in the magnitude of forces results in decreased cell-mediated contractions^10^. Furthermore, the release of mechanical forces also results in decreased of contractility of myofibroblasts by inhibition of TGF-β1 activation^76^. The PPI network for DEGs at 7 days of TE showed that *ACTN2*, followed by *ALDOC,* are the most crucial hub genes in this network, which consisted of modules associated with metabolism, muscle contraction, ECM remodeling, cell adhesion and immune response. The importance of decreased expression of cytoskeletal genes, including *Actn2* and *Tnnt3,* for inhibition of myofibroblast activity was shown in a murine fibrosis model by treatment with carbon monoxide^77^. Thus, correlating the observed molecular changes with pressure measurements during TE would provide valuable information about how cells’ adaptation to the external tensions reduces overall pressure applied on the skin. KEEG analysis at 7 days of TE also indicated a potentially important role of the HIF-1 signaling pathway in cells’ adaptation to environmental changes in tissue remodeling at later stages of TE. The crucial role of HIF-1α has been reported in diverse biological processes, including neovascularization, angiogenesis, cytoskeleton organization, and energy production^78–80^. In line with this result, we reported changes in expression of HIF-1 target genes, including an increase in *ID2* and a decrease in *ALDOC* and *LEP* expression between 24 h and 7 days of TE^80^. These genes encode proteins involved in the regulation of keratinocyte proliferation and cell metabolism^80–82^.

## Materials and methods

### Experimental design

TE were performed on 5–6 weeks old female Yucatan mini pigs to mimic clinical setting. One week before the procedure to place the tissue expanders, four 10 cm by 10 cm grids were tattooed on the back of the animal. The 120 cc rectangular tissue expanders (PMT Corporation) were inserted subcutaneously underneath two of the four grids, one anteriorly over the ribs, one posteriorly over the abdomen, and the untouched contralateral sides served as controls. After 14–18 days of recovery, the tissue expanders were injected with 60 ml of saline to begin TE, and the skin covering the expanders sustained the expansion for 1 h (TE.1h), 24 h (TE.24h), 3 days (TE.3d) or 7 days (TE.7d). Next, three-mm skin punch-biopsy samples were collected in triplicates from the apex (“a”) and middle (“b”) of the tissue from each expanded grid, as well as from the corresponding control sides. All animals were euthanized immediately after the tissue harvesting. Figure 1a illustrates the experimental design and timeline.

### Total RNA extraction and quantification

For each tested condition (TE.1h, TE.24h, TE.3d, TE.7d), full-thickness skin biopsies were collected in triplicates from the apex (“a”) and middle (“b”) of the expanded tissues. Control skin biopsies were collected from contralateral unexpanded sites. Total RNA was extracted using RNeasy Mini kit (Qiagen), followed by on-column DNAse I digestion, according to manufacturer recommendations. The concentration was measured using a Qubit fluorometer (Invitrogen). The quality was evaluated using 1% agarose gel and Nanodrop Spectrophotometers (ThermoScientific). For Next Generation Sequencing (NGS), triplicates of RNA samples from each category were combined by mixing equimolar concentrations of each, giving two RNA sample pools (“a”, “b”) per each timepoint and eight RNA control pools (total 16 RNA pools). Samples “a” and “b” for each timepoint were treated as replicates.

### mRNA library construction and sequencing

The paired-end RNA-seq analysis was performed by BGI (https://www.bgi.com/global/) using Illumina HiSeq 4000. Low-quality reads containing adaptors and a high content (> 5%) of unknown bases were removed from the analysis. The mapping RNA-seq reads were performed using HISAT program (v0.1.6-beta). Alignment of the sequencing reads to a reference sequence was performed using Bowtie2 (v2.2.5) and gene expression level was calculated with RSEM software package (v1.2.12). mRNAs that were not detected in any sample (read count less than 1) were excluded in the downstream analysis.

### Differential expression analysis

Differential expression analysis was performed using R v3.6.0 with the Bioconductor-DESeq2 package v1.26.0. Results were corrected for multiple testing using the Benjamini–Hochberg method and reported as an adjusted *p*-value. A combination of an adjusted *p*-value < 0.05 and absolute log_2_ fold change (|log_2_(FC)|) value > 1 were used as the threshold to determine the significance of differentially expressed genes (DEGs). Volcano plots of DEGs were prepared using R and “*EnhancedVolcano*” library.

### Sample distance and principal component analysis (PCA)

The overall similarity between samples was evaluated by the Euclidean distance calculated using the R function “*dist*” on the rlog-transformed data and unsupervised hierarchical clustering, and by PCA analysis using the “*plotPCA*” built-in DESeq2 function.

#### Real-time PCR analysis (qRT-PCR)

Synthesis of cDNA was performed from 200 ng of total RNA using the High-capacity cDNA Reverse Transcription kit (Life Technologies) according to the manufacturer’s recommendations. The qRT-PCR was performed on StepOnePlus Real-Time PCR system (Applied Biosystems) using TaqMan Assay (Applied Biosystems). Cycling conditions included an initial denaturation at 95 °C for 15 min, then 45 cycles at 95 °C for 15 s and 60 °C for 1 min. The cycle quantification values (Cq) were calculated using the threshold cycle method. Fold change of gene expression was calculated with the 2^−ΔΔCT^ method^83^ using *B2M* reference gene.

### Pathway enrichment analysis

The functional enrichment analysis was performed using the g:GOSt functional profiling tool available on the g:Profiler web server (version e99_eg46_p14_f929183) with the Bonferroni multiple testing correction method, applying a significance threshold of 0.05 (https://biit.cs.ut.ee/gprofiler/)^84,85^. Gene Ontology (GO), Kyoto Encyclopedia of Genes and Genomes (KEGG) and Reactome (REAC) databases were used as sources and electronic GO annotations were excluded to increase the accuracy of the results. The obtained results were visualized in R using the “*ggplot2*” library to create dot plots graphs, and in Cytoscape software v3.7.2 using the EnrichmentMap and AutoAnnotate applications.

### The construction of the time-related protein–protein interaction networks in TE

The protein–protein interaction (PPI) networks associated with progressive molecular changes during TE were constructed using the Search Tool for the Retrieval of Interacting Genes database (STRING v11)^86^. DEGs corresponding to different time points of TE were used as input. We selected only interactions derived from experiments and databases at a high level of confidence (sources: experiments, databases; score ≥ 0.7). The Cytoscape software v3.7.2 was used to visualize and analyze the PPI network. The Molecular Complex Detection (MCODE) tool was used to search for high modularity clusters within the network (degree cutoff = 2, node score cutoff = 0.2, k-score = 2, maximum depth = 100) and modules which consisted of at least six nodes and had an MCODE score of at least of 4 were considered significant. The PPI network was analyzed by NetworkAnalyser and Centiscape v2.2 built-in Cytoscape applications, and nodes with degree, closeness centralities and betweenness centralities scores higher than the mean values were classified as the hub genes. All nodes not connected to the main network were excluded from this analysis to obtain reliable results.

### Ethics statement

Our study had been approved by Northwestern University Institutional Animal Care and Use Committee (IACUC #LCH14-006) and complied with NIH standards provided in the “Guide for the Care and Use of Laboratory Animals”. All animal procedures were performed according to approved protocol.

## Supplementary information

Supplementary Figure

Supplementary Tables

## Data Availability

The raw sequencing reads of all libraries have been deposited at GeneBank (SRA accession: PRJNA639589; SUB7598242) and can be found at https://www.ncbi.nlm.nih.gov/sra/PRJNA639589 after the release date.
