# Supplementary figures and images for: Transcriptomic analysis reveals dynamic molecular changes in skin induced by mechanical forces secondary to tissue expansion
Source: Sci Rep. 2020 Sep 29;10:15991. doi: 10.1038/s41598-020-71823-z (PMC7524724; doi:10.1038/s41598-020-71823-z)

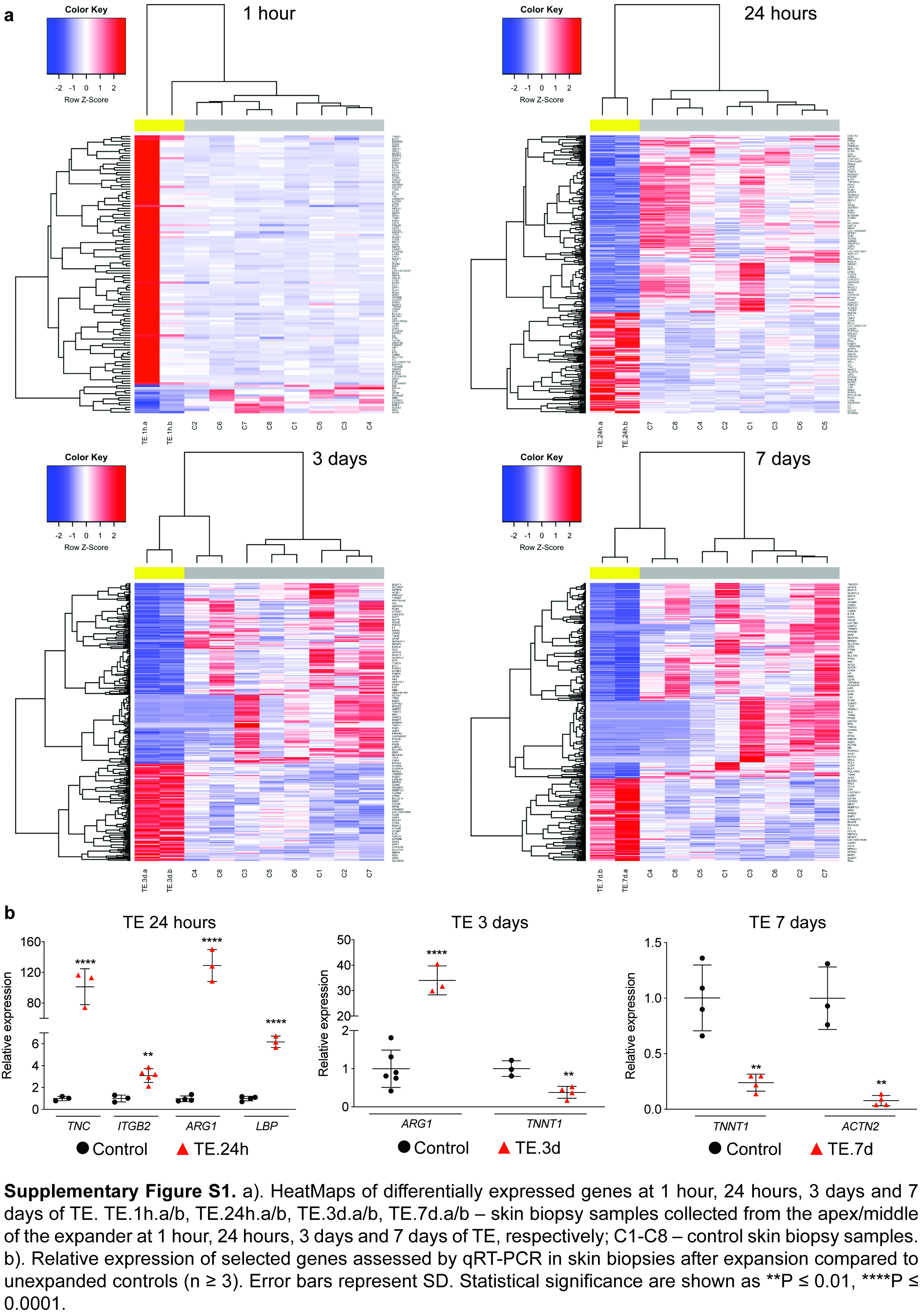

Supplement: Supplementary file 1 — Supplementary Figure [file 41598_2020_71823_MOESM1_ESM.tif]
